# Management of onchocerciasis among adolescents in nkwanta north district of Ghana: Qualitative study of adolescents’ perception, community and health system support

**DOI:** 10.1371/journal.pntd.0011577

**Published:** 2023-08-29

**Authors:** Sitsofe Gbogbo, Hubert Amu, Robert Kokou Dowou, Martin Amogre Ayanore

**Affiliations:** 1 Department of Population and Behavioural Sciences, School of Public Health, University of Health and Allied Science, Hohoe, Ghana; 2 Department of Epidemiology and Biostatistics, School of Public Health, University of Health and Allied Science, Hohoe, Ghana; 3 Health Policy Planning and Management, University of Health and Allied Science, Hohoe, Ghana; Federal University of Agriculture Abeokuta, NIGERIA

## Abstract

**Background:**

Onchocerciasis affects the quality of life to a greater extent among affected individuals. The World Health Organization (WHO)’s Onchocerciasis Control Program (OCP) has effectively reduced the prevalence of onchocerciasis by interrupting the transmission of the parasite and by mass population treatment in the regions at risk of the disease. Despite the successful reduction of the prevalence of onchocerciasis by WHO, the socioeconomic burden resulting from the disabilities caused by onchocerciasis are still immense. This study sought to explore the adolescents’ perception regarding the management of onchocerciasis, community and health system support in Nkwanta North District of Ghana.

**Method:**

This study adopted a qualitative phenomenological design and exploratory, descriptive qualitative approach. An in-depth interview guide was developed to collect data for the study. One-on-one interview was conducted. Data collected from 16 onchocerciasis adolescent patients were analysed thematically using ATLAS.ti v7.5.7. Quotes from the participants were presented verbatim to substantiate the themes realised.

**Results:**

Most of the 12 participants (75.0%) were aged 15–18 years old. It was noted that 6(37.50%) of participants were in Junior High School (JHS), while 4(25.0%) were in Senior High School (SHS). It was noted that community members have diverse understandings and perceptions of onchocerciasis, including beliefs that Onchocerciasis is a serious disease that can cause blindness; it is caused by the consumption of some types of food products or stressful work. Adolescents believed that onchocerciasis was caused by insect bite blood infection, poor environmental hygiene, sun or could have been inherited from parents. Ivermectin treatment was noted by adolescents to have helped relieve the symptoms of ochocerciasis they were experiencing. However, the adolescents indicated that they had experienced some side effects, including fever, headache, body itching, rushes, swollen body and blurred vision from the drug.

**Conclusion:**

Inadequate education and communication about onchocerciasis resulted in diverse and erroneous meanings of onchocerciasis among community members. Our research recognises that community and health system supports is very important in the effective management of Onchocerciasis, contributing to the attainment of Sustainable Development Goal (SDG) 3.3, which is targeted at ending the epidemic of NTDs like onchocerciasis by 2030.

## Author summary

Our study explored the management of onchocerciasis among adolescents, perception, community, and health system support in Nkwanta North district of Ghana. Our results revealed that community members have various perceptions of the mode of transmission and the risk factors of onchocerciasis. For example, some community members believe that onchocerciasis is a very serious disease which if you get can make you blind. Others believe that onchocerciasis is caused by the consumption of some types of food products or stressful work. Adolescents on the other hand believed that onchocerciasis was caused by insect bite blood infection, poor environmental hygiene, sun, or could have been inherited from parents. The adolescents reported indicated that Ivermectin treatment had a great positive impact on their condition by helping relieve symptoms they were experiencing. We found that community support available for adolescents’ management of onchocerciasis was financial and emotional support. Also, we found that financial support, free treatment, home visit, counseling, and education were health system supports available to adolescents for the management of their condition. We concluded that community-based education should be provided to communities by health authorities to educate the communities on NTDs because this will help address the diverse and erroneous perceptions.

## Introduction

Onchocerciasis is a neglected tropical disease (NTD) caused by the nematode Onchocerca volvulus. Onchocerca volvulus is transmitted through the bites of blackflies of the genus Similium that breed near rivers and streams in regions with constantly moving water [1]. S. damnosum complex and S. neavei found in Africa and the Middle East and in parts of East Africa respectively are the most important vectors. However, in America, the most important vectors are S. ochraceum, S. metallicum, S. oyapockense, S. guianense, and S. exiguum [2]. Although this disease occurs mostly in warm tropical settings, its parasitic flies survive under environments favourable for their growth all year round [3]. The disease is known to affect rural populations and is a major cause of blindness and Onchocercal skin disease (OSD) in endemic areas with severe socioeconomic outcomes [4]. Symptoms include severe itching, disfiguring skin conditions, and visual impairment [5]. Globally, it is estimated that about 90 million people are at risk of being infected with Onchocerca volvulus in endemic areas mostly in sub-Saharan Africa (SSA), and out of which about 37 million are infected and 300, 000 are permanently blind as a result of onchocerciasis [1]. However, 1 to 5 percent of the population in onchocerciasis-endemic areas who are exposed to a high rate of infection transmission do not exhibit any clinical signs of the disease and are thus considered putatively immune individuals [6]. It is known that onchocerciasis is a chronic, slowly progressive, parasitic disease that has been regarded as the second-leading communicable cause of blindness globally with around 500,000 persons blind yearly [7]. A study by Crump, Morel, and Omura [8] revealed that, out of approximately 123 million persons who are at risk for infection in 38 endemic countries, 25.7 million are infected and 1 million are blinded or have a serious visual impairment. Onchocerciasis is endemic in SSA countries causing partial or total loss of sight and extreme itching with skin conditions [9] as a result of high infection load [10]. Study shows that onchocerciasis is the second most common infectious cause of avoidable blindness [11]. These have a significant impact on people’s social and economic lives, which lead to poverty and impede their development [11]. All because, they become unproductive, and those who are badly afflicted spend all of their time and money roughly $20 per year, or 15% of their yearly income on their health and medical treatment [12]. It was estimated in 2017 that 99 percent out of the estimated 20.8 million onchocerciasis cases came from the poorest and most vulnerable populations of SSA [13]. Out of the 218 million people, about 25 million people are infected globally and 90 million people are highly at risk of the disease with more than 99% of the cases coming from 31 countries in the SSA [14]. Of those who are infected, 14.6 million suffer from skin disease and 1.15 million population has been estimated to experience loss of sight while 220,000 are confirmed to be completely blind which caused 1, 136,000 Disability Adjusted Life Years (DALYs) in 2015 [14–17] and 1.23 million DALYs in [17]. The WHO’s Onchocerciasis Control Program (OCP) has effectively reduced the prevalence of onchocerciasis by interrupting the transmission of the parasite and by mass population treatment in the regions at risk of the disease [7]. The widespread use of ivermectin therapy is essential for managing and eradicating onchocerciasis as a public health issue, which will help to stop the pandemic of neglected tropical diseases by 2030 [15]. From WHO report, efforts to stop the spread of the disease have improved as of 2020, but six countries saw a 27% reduction in coverage as a result of their inability to carry out large-scale treatment plans, which was made worse when the covid pandemic first appeared [15]. In response, several studies contend that community involvement is essential for managing and eradicating onchocerciasis or even all NTDs, particularly in areas with limited resources [18]. Additionally, the health system has implemented test-and-treat (TNT) procedures, which are meant to identify patients who require therapy, need to stop receiving it due to side effects, or do not require it [19]. Ivermectin Mass Drug Administration (MDA) remains the primary method for controlling with effort of eradicating onchocerciasis in Ghana [20].

Despite several years of MDA with ivermectin, infection with onchocerciasis and the commonly associated clinical manifestations of the disease still persist in many regions and districts across Ghana [20]. Despite the significant reduction in the prevalence of onchocerciasis in Ghana, there are still communities with positive microfilaria prevalence above 1% [21]. For instance, a study carried out in three communities (Tanfiano, Senya and Kokompe) in Ghana showed that the prevalence of microfilaria in the communities were 13.2, 2.4, and 2.9%, respectively [20]. Adolescents are among the school-going children that are targeted by the Ghana Health Service for MDA program and other interventions that are targeted toward the elimination of onchocerciasis in Ghana because adolescents are among the highest group at risk of onchocerciasis in the Nkwanta North District of Oti region. Despite these efforts, recent observations within the district show that more and more adolescents are diagnosed and treated for onchocerciasis. There are limited scientific studies documenting adolescents’perceptions regarding the management of onchocerciasis and the support that is available to them. In this view this study sought to explore the adolescents’ perception regarding the management of onchocerciasis, community and health system support in Nkwanta North District of Ghana.

## Methods

### Ethical approval and consent to participate

This study was approved by the University of Health and Allied Sciences Review Committee (**Reference number: UHAS-REC A.8l4l2t-22**). Consent to participate in the study was also obtained through written informed consent forms and parental assent forms from the participants and their parents. All methods were carried out following relevant guidelines and regulations.

The reporting of this study is guided by the consolidated criteria for reporting qualitative research (COREQ) [20].

### Setting

The study was conducted in Nkwanta North District of the Oti Region, Ghana. The district is located in the north-eastern part of Ghana. The district is located between Latitude 7°30’N and 8°45’N and Longitude 0°10’W and 045’E [22]. The District Capital, Kpassa is located 270 km to the South of Ho (the Regional Capital). The population of Nkwanta North District, according to the 2010 Population and Housing Census, is 64,553 representing 3.0 percent of the region’s total population [22]. The Nkwanta North District is one of the districts in the Oti Region with the highest level of onchocerciasis prevalence and adolescents are most at risk of the infection [23].

### Design

This study adopted a qualitative phenomenological design and exploratory descriptive qualitative approach. This design and approach were adopted in this stusy to facilitate an in-depth exploration of adolescents’ illness experiences of onchocerciasis. The phenomenological design was used in this study because we sought to hear adolescents affected by onchocerciasis tell their experiences about living with onchocerciasis. This gave us an in-depth understanding of their perceptions of the condition and its management using both routine MDA and TNT procedures [24] using in-depth interviews.

### Study population

The main target population for the study was adolescents between 10 and 19 years old who were affected by onchocerciasis in the Nkwanta North District at the time of data collection for the study.

### Sampling

The purposive and snowball sampling strategies were used in selecting participants for this study. The adolescents were selected based on whether they still have the disease and are on treatment or have just completed treatment. The adolescents who have completed their treatment within one year of the time of this study were selected because they can provide rich information on the issues being studied. They were chosen on the basis that they represent a specific perspective on the phenomenon rather than a population [25]. Gatekeepers and key informants such as chiefs, queen mothers, assembly members, Nurses, and public health officers were used as a means of gaining access to the adolescents.

### Sample size

We sampled 16 adolescents for the conduct of this study. Our sample size was based on data saturation.

### Data collection

Data were collected using in-depth interviews in collecting data from the participants through face-to-face interactions. Data for this study were collected using an in-depth interview guide. A structured data collection tool was used to collect data on the socio-demographic characteristics of the participants. The interviews were conducted in the homes of the participants. The interviews were conducted in English and Ghanaian languages (Ewe, Dangoba, and Twi). The choice of local language for the conduct of the interviews was to ensure that we have in-depth information without any language barrier challenges. To ensure that the use of the local languages did not affect the quality of data collected, the researchers who could not speak the local language were assisted by interpreters that speak the local language fluently. All interviews were recorded using a digital audio recording device, permission was obtained from all participants to record each interview. Hand-written notes were taken during the interview process to record responses given by the participants. The purpose of using both the audio recorder and hand-written notes is to ensure that the interview process is not halted should any of the equipment (pen or audio recorder) breakdowns during the interview process. To ensure the trustworthiness of the instrument, interview guides were given to experts in qualitative research to peruse the appropriateness of the questions before we used them.

### Ethical considerations

Ethical clearance (**Reference number: UHAS-REC A.8l4l2t-22**) was obtained from the University of Health and Allied Sciences Review Committee and permission was sought from the district assembly and district health directorate before data was collected. Provision was made for the translation and interpretation of the instrument into Ewe and other local languages that are mostly understood and spoken by a large proportion of people in the study setting. Written informed consent was obtained from participants who were above 18 years. However, for participants who were below 18 years and were still minors during the time of the data collection, both individual from the adolescents and parental consents were sought before adolescents were interviewed. This had been done by giving them informed consent and parental ascent form forms to sign, indicating their willingness to participate in the study. For those adolescents whose parents were not available during the data collection; we obtained their consent and ascent of their respective guardians before their participation in the study. Participation was purely voluntary and they were informed of their right to withdraw from the study at any time. This had been done by giving them written informed consent and parental assent forms to the parents or guardians to sign, indicating their willingness to participate in the study. The purpose of the study was also explained to the parents/guardians and also to the adolescents themselves before interviewing them. They were informed of their right to stop at any point in time of the interview process when they feel so and also assured that any statement or comments including that of privacy will remain protected after the data collection. Participants and their parents/guardians were identified and approached with the help of key informants (Nurses and public health officers). Since the interviews were carried out in the home of the participants, their parents were informed about the study by visiting them in their homes. All the parents and the participants voluntarily agreed to participate in the study. The confidentiality of the participants was adhered to by ensuring that no information from participants was disclosed to any third party. The respondents were identified by codes and numbers instead of their real names. All the interviews were conducted by qualitative researchers with the support of research assistants who were well-trained in qualitative data collection. The computer which was used for the transcription of interviews was protected with passwords.

### Data analysis

All recorded interviews were transcribed verbatim into a word document in English before codes and themes were developed thematically using ATLAS.ti. v7.5. Transcripts were analysed thematically [25]. Familiarization with the data was done to take note of key ideas and recurrent themes. Coding was done based on the research objectives as well as themes that emerged from the data itself. Quotes from the participants were used in presenting the data to substantiate issues discussed by participants. The initial themes that were developed were reviewed and refined into final themes taking into consideration internal homogeneity and external heterogeneity [26]. The themes were defined and named and a detailed analysis was conducted and written based on how they fit into the broader story of the data. A frequency table was, however, used to present the socio-demographic characteristics of the participants.

## Results

### Participants’ profile

**Table 1** presents the profile of the participants. We noted that 12 (75%) of the participants were aged 15–18 years old. Regarding the educational status of the participants, we noted that most of them 6(37.50%) were still in Junior High School (JHS) while 4(25.0%) were in Senior High School (SHS). Approximately, 6(37.50%) of the participants had lived with the condition (Onchocerciasis) for 1–5 years and 6–10 years respectively. However, 4(25.0%) of them had lived with the condition for more than 10 years.

**Table 1:** Socio-Demographic Characteristics of Participants.

Variables	Frequency (%)	Percentage (%)
Age (in years)		
10–15	2	12.5
15–18	12	75.0
>18	2	12.5
Sex		
Female	8	50.0
Male	8	50.0
Ethnicity		
Mole-Dagbani	16	100
Marital status		
Never married	16	100
Level of Education		
None	4	25.0
Primary	2	12.5
JHS	6	37.5
SHS	4	25.0
Religion		
Christianity	13	81.3
Islam	1	6.2
Africa Traditional	2	12.5
Occupation		
Student	11	68.7
Farmer	3	18.8
Trader	2	12.5
Duration of living with condition (in years)		
1–5	6	37.5
6–10	6	37.5
10 and more	4	25.0
**Total**	**16**	**100**

### Thematic results

#### Adolescents perceptions of mode of transmission and risk factors of onchocerciasis and its management

**Table 2** presents the themes derived from the interviews conducted among onchocerciasis adolescents on the meaning created by onchocerciasis. Four main themes were realized. These were; Community perception of the mode of transmission and risk factors of Onchocerciasis, adolescents perception of the mode of transmission and risk factors of Onchocerciasis, adolescents’ perceptions of ivermectin treatment of onchocerciasis and adolescents’ experience regarding Ivermectin treatment.

Regarding perception of the mode of transmission and risk factors of Onchocerciasis, most of the participants indicated that the community members have diverse perceptions regarding onchocerciasis. According to the adolescents, the community members believe that onchocerciasis is a serious disease that can cause blindness. Adolescents further stated that most of the community members believe that it is caused by the bite of insects while some of them also believe that it is caused by the consumption of some types of food products or stressful work (farming). The following quotes summarised some of the responses from the participants

“*People say the condition can lead to eye blindness and there will be some worms on your eyes when you have such a condition*. *The condition mostly affects the eyes”*

Female Participant, 18 years

“*Some people say that there are types of food which are not supposed to be eaten and once eaten*, *you will get the disease*.”

Male Participant, 18 years

“*Most of the people in my community said the condition was a result of the farming activity I engaged myself in because it is stressful work”*.

Female Participant, 16 years

“*People say you can get the disease through insect bites*, *especially at the farm*.*”*

Male Participant, 19 years

Concerning the adolescents’ perception of the mode of transmission and risk factors of Onchocerciasis; most of the adolescents believed that onchocerciasis is caused by insect bites, blood infection [genetic infection], poor environmental hygiene, and sun. Also, others noted the disease could have been inherited from parents. The following quotes summarise some of the views of the adolescents:

“*I know the disease is hidden in some foods*, *so once we eat that food*, *we get infected*. *Food like maize has to be milled and mixed with another food item to prepare food*, *so the infection can be hidden in such foods*, *and once eaten*, *you get the disease*. *Also*, *when you go to the farm*, *insects like mosquitos and millipede and centipede can bite you and give you the disease*.*”*

Male Participant, 18 years

“*What I know is that the disease affects the eyes and I can become blind*, *and I also believe when you stay under the sun for long …”*

Female Participant, 18 years

“*When I was growing up*, *I realized that there was something like a ball on my father’s body*, *also*, *I have seen that something on my body as well*. *I think the condition is something in the blood because my father was also having it*.*"*

Male Participant, 18 years

Vis-à-vis of the adolescents’ perception of the ivermectin treatment as part of the management of the condition, most of the adolescents specified in their interviews that the ivermectin treatment has led to positive change in the condition by helping relieve the symptoms they were previously experiencing. The following quotes represented the views of some of the adolescents:

“*The drug has helped me a lot the pains I used to feel in my body and joints are no more after I took that drug*. *It has helped me because all the challenges I have by then were all resolved after I took the drug*.*”*

Male Participant, 18 years

“*The drugs are good and I experienced a positive change in my condition after taking the drugs*. *The pains I felt became normal as compared to the previous time*.*”*

Female Participant, 16 years

“*Before I was admitted to the health facility*, *I used to experience fever*, *headache*, *and blurred vision but all these went down when I took the medicine and I did not experience any side effects after taking the medication*, *I felt well and normal now*.*”*

Male Participant, 19 years

“*I don’t know what others think of the medicine but for me*, *per my experience*, *I never experienced any side effects when I take the medicine*. *Before admitting me to the health facility*, *I used to experience fever (high temperature)*, *headache*, *and blurred vision but all these went down when I took the medicine*. *However*, *with the fever and headache*, *I still experience it because I work in the sun*.*”*

Male Participant, 18 years

Concerning the adolescents’ experiences regarding Ivermectin use, all the participants indicated that after the use of Ivermectin, they experienced, mild to severe side effects including fever, headache, body itching, rushes, swollen body, and blurred vision. The following quotes summarise some of the views of the adolescents:

“*When I took the medicine the whole of my body became swollen*, *and my body itches but that only lasted for some time*, *the pains I felt became normal and I can now see well as compared to the previous time…”*

Female Participant, 18 years

“*After I took the medicine I had some side effects*, *my body was itching me*, *and I felt like I was getting blind but along the line the blurred vision*, *the headache*, *and others went down”*

Female Participant, 17 years

“*My body became swollen after I took the drug but the drug has helped me a lot*. *It has helped me because all the challenges I have by then were all resolved after I took the drug*.*”*

Female Participant, 19 years

“*After taking the medicine some rushes came on my body and my body was itching and my body became a little big*, *but the drug was helpful*.*”*

Male Participant, 18 years

**Table 2:** Meaning created towards Onchocerciasis and its management.

Themes	Sub-themes
Community’s perceptions of mode of transmission and risk factors of onchocerciasis	➢ Disease is serious ✓ cause blindness ➢ disease could have been caused by some types of food ➢ disease could have been stressful work (farming) ➢ disease caused by insects bite
Adolescents’ perceptions of the mode of transmission and risk factors of onchocerciasis	• Disease is caused by: ✓ Insects bite ✓ Blood infection ✓ Poor environmental hygiene ✓ Inherited ✓ Sun
Adolescents’ perceptions of ivermectin treatment of onchocerciasis	Helps relieve symptoms (Pain)
Experience regarding Ivermectin use	• Side effects ✓ Body itching ✓ Swollen body ✓ blurred vision ✓ fever ✓ headache ✓ Rushes Symptoms relief

### Community and health system support

**Table 3** presents the themes derived from the interviews conducted among adolescents affected by onchocerciasis on community and health system support available for them during the management of their condition. Two themes emerged from the interviews: community-based support and health system support.

Regarding the community-based support available for adolescents, we noted that the community support available for the management of their condition mostly comes from family and relatives and the churches. Most of the adolescents also noted that they get support from church members, such as regular visits, encouragement, and prayers. This support serves as a moral boost and a coping mechanism for soothing adolescents from the pain they might have been going through at a particular moment. The following quotes represent the views of some of the adolescents:

“*Yes*, *my church members from the church where I and my parents worship came to check up on me on my return from the health facility*.*”*

Female Participant, 16 years

“*Yes*, *I had support from my church people*. *Some gave me money and other things*, *and some also pray for me when they came to our house to visit me*.*”*

Male Participant, 15 years

The family support available for the adolescents as described in their interviews includes financial and social support (fetching water, cooking).

“*My mother was helping me with water and other things I needed*, *my pastor and church members also prayed for me and some of my friends also came to visit me*.*”*

Male Participant, 19 years

“*My father helping me with everything and my sister also has been giving me money to be managing myself and to take me to school when I came back from the hospital*.*”*

Female Participant, 15 years

Concerning the Health system-based supports, we noted the main supports that were available to the adolescents in the management of their condition included financial support, free treatment, a home visit (check-ups), counseling, and education.

Regarding the financial support, the participants indicated that the facilities that took care of them gave them money to take care of themselves when they get home. Financial support for the adolescents could be a way to encourage other infected people who have not reported to health facilities for treatment to seek care. The following are some of their views:

“*…I was given money to use to support ourselves for the time we have spent at the hospital*.*”*

Male Participant, 15 years

“*For example*, *they gave me some amount of money to use to support myself when I get back home and for my time spent at the health facility leaving things I could have been doing when home…”*

Female Participant, 19 years

“*… they transported us to the health facility and they gave us some money to use to support our farm work for the time we have spent there leaving our farm…”*

Male Participant, 18 years

Concerning the free treatment, the adolescents reported in their interviews that all the cost for the treatment of their condition (Onchocerciasis) was borne by the health facility without them paying any fees.

“*They always go around and dress our operated wounds for us explaining things to us about how to care for ourselves when we get back home*, *they did all these for free*, *I did not pay any money for my treatment…”*

Male Participant, 18 years

“*When we went to the hospital*, *the doctors did not take any money from us*, *and the drugs and operation were free of any charge*, *which was very helpful to us”*

Female Participant, 15 years

Furthermore, the adolescents indicated that when they were at the facility, there were health professionals that specifically counseled and educated them on what to do or not to do so to get better faster and become healthier.

“*The doctor was going around and dressed our operated wound for us*, *educated us*, *and counsel us not to do any hard work till the wound is healed and also not to take drugs unnecessarily*.*”*

Female Participant, 17 years

“*Yes*, *they educated us and asked us not to buy any medicine on our own when we are not feeling well but we should rather go to the hospital with the small card they gave us so that they will take care of us instead in other to prevent any complications*.”

Male Participant, 19 years

The interviews also showed that home visit was one strong part of the health system support which is available for adolescents affected by onchocerciasis. Some of the participants have these to say:

“*when I got home after the operation the doctor was still coming to my house to help dress my wounds till it got healed*.*”*

Female Participant, 16 years

“*I was not given any medicines again but there was a doctor who normally comes around to dress my wounds in my house*, *if he will not come himself*, *there is a nurse that will come to do it for me in the house*.*”*

Female Participant, 19 years

“*When I came back from the hospital*, *one of the nurses has been coming from time to time in my house to treat the wound and also make me take medicine*.*”*

Male Participant, 19 years

**Table 3:** COMMUNITY AND HEALTH SYSTEM SUPPORT.

Community-based supports	➢ Church support ✓ Visit ✓ Prayers ➢ Family support ✓ Financial ✓ Social support (fetching water, cooking) ✓ Prayers
Health system-based supports	• Financial support • Free treatment • Free transport to the health facility • Counseling • Education • Home visit

## Discussion

In our study, we explored the management of onchocerciasis among adolescents, perception, community, and health system support in Nkwanta North district of Ghana. We noted in this current study that community members have diverse perceptions of the mode of transmission and the risk factors of onchocerciasis. Some community members believe that onchocerciasis is a serious disease that can cause blindness, and some believe that it is caused by the consumption of some types of food products or stressful work. Adolescents themselves believed that onchocerciasis was caused by insect bite blood infection, poor environmental hygiene, sun, or could have been inherited from parents. These findings are consistent with previous studies [27–31]. The findings from our study where the adolescents reported that the community members believed that onchocerciasis is caused by consumption of food products or stressful work at the farm, poor environmental hygiene, sun or is inherited from parents at birth could be attributed to inadequate health education and information about NTDs among the general populaces especial those in the remote areas of the district where the mode of communication and information are limited [32–34]. These findings imply that more health education campaigns, including using community communication systems on onchocerciasis and other NTDs, is imperative to correct the misunderstandings or misperceptions of communities of the mode of transmission and the risk factors of Onchocerciasis. This is very important because people ‘s correct understanding of how diseases like onchocerciasis os transmitted could be crucial in their effort to take measures to prevent it.

Concerning Ivermectin treatment, the adolescents reported in this study that it had a great positive impact on their condition as it had helped relieve symptoms they were experiencing before the initiation of the treatment. These findings are unswerving from the reports by the previous studies [35–37]. However, some of the adolescents also indicated that they had experienced some side effects, including fever, headache, body itching, rushes, swollen body, and blurred vision from the drug. These findings are consistent with the reports of the previous studies [38–41]. These experience of side effects could discourage some adolescents from taking the Ivermectin treatment or participating in the MDA programm,e that is one of the major strategies to eradicating onchocerciasis in Ghana. Therefore it is important health workers educate or inform the communities about the possible side effects of the Ivermectin treatment so that the community members who have received the treatment are not surprised by these side effects if they experience them.

We found that the community support available for the management of onchocerciasis among adolescents was financial and emotional support (encouragement and prayers), and these supports mostly come from family and relatives and church members. These supports were considered by the patients to have been very crucial during the treatment of their condition and recovery afterwards. Prayers, encouragement, and counselling from family and church members were reported by adolescents to have helped them emotionally and strengthened them to go through whatever pain they might have been experiencing as a result of the condition and also the anxiety and depression as a result of stigmatisation [18, 42–46]. This point to the protective factor of social support to people affected by onchocerciasis NTDs. Hence, it is important more steps are taken by programme planners and implimentaters to include education on social support into the interventions that are targeted at elimating or eradicating NTDs to educate communties on the importance of social support to the patients living with any of NTDs. This if well done could help maximise the level of community support among the communities and also help reduce the stigmatization.

In this current study, we found that financial support, free treatment, home visit, counseling, and education were health system supports available to adolescents who suffered from onchocerciasis. These supports especially the financial and free treatment from the health system which were widely reported and appreciated by the adolescents could be ascribed to the fact that onchocerciasis, as one of the major NTDs, is known to disproportionately affect the most disadvantaged: the rural poor with reduced access to health care services, therefore free treatment was very important for the management of their condition and also fight the elimination of onchocerciasis [5, 47–51].

### Strength and weakness

The current study was a community-based study conducted in Nkwanta North District using an in-depth understanding of the lived experiences and the perceptions of adolescents of the mode of transmission of Onchocerciasis and its management. This has helped to explore the well-detailed scope of how patients infected with onchocerciasis live with their condition and how they effectively cope with daily life experiences.

Despite the strength of this study, it also has some limitations. The use of in-depth interviews for the data collection in this research project could have introduced response bias on the part of the participants. The use of purposive as well as snowball sampling introduced the possibility of selection bias on the part of the data collectors. There might also be recall bias from the participants and social desirability bias on the requested data. The use of different languages (English and local languages) and also the use of interpreters to collect the data could have distorted the presentation of the questions to the participants.

## Conclusion

We found from the study that community members have diverse perceptions about onchocerciasis. Adolescents believed that onchocerciasis was caused by insect bites, poor environmental hygiene, or could have been inherited from parents. We noted in this study that adolescents believe that the Ivermectin treatment could have had a great positive impact on the condition as it has helped relieve the symptoms they were experiencing before the initiation of the treatment. Our research recognises that community and health system supports is very important in the effective management of onchocerciasis, contributing to the attainment of Sustainable Development Goal 3.3, which is targeted at ending the epidemic of NTDs like onchocerciasis by 2030. These findings call for more community-based education by health authorities to educate the communities on NTDs and how they are transmitted. As it could help address the diverse and erroneous perceptions the adolescents and the communities have of the onchocerciasis mode of transmission. Also, further measures need to be taken by Ghana Health Service to include educational programmes on social support in the interventions that are targeted at eliminating or eradicating NTDs to educate communities on the importance of social support to the patients affected by any of NTDs.
